# *catena*-Poly[[aqua­bis­[2-(3-benzoyl­phen­yl)propano­ato-κ^2^ *O* ^1^,*O* ^1′^]cadmium(II)]-μ-4,4′-bipyridine-κ^2^ *N*:*N*′]

**DOI:** 10.1107/S1600536811001280

**Published:** 2011-01-15

**Authors:** Tao Jiang, Seik Weng Ng

**Affiliations:** a Department of Food and Environmental Engineering, Heilongjiang East University, Harbin 150086, People’s Republic of China; b Department of Chemistry, University of Malaya, 50603 Kuala Lumpur, Malaysia

## Abstract

The 4,4′-bipyridine heterocycle in the polymeric title compound, [Cd(C_16_H_13_O_3_)_2_(C_10_H_8_N_2_)(H_2_O)]_*n*_, links adjacent Cd(II) ions  into a chain running along the *c* axis. The Cd atom, which lies on a twofold rotation axis, is chelated by the carboxyl­ate unit and exists in a seven-coordinate penta­gonal–bipyramidal geometry. The apical sites are occupied by N atoms. The water mol­ecule also lies on the twofold rotation axis. The methyl substituent of the propano­ate group is disordered over two positions in a 1:1 ratio. O—H⋯O hydrogen bonding between water molecules and adjacent carboxylate O atoms is observed.

## Related literature

For the crystal structure of the parent carb­oxy­lic acid, see: Briard & Rossi (1990 ▶). For related metal carboxyl­ates, see: Zhang *et al.* (2007*a* ▶,*b* ▶).
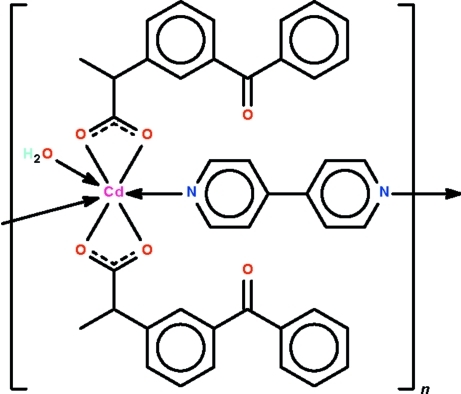


## Experimental

### 

#### Crystal data

[Cd(C_16_H_13_O_3_)_2_(C_10_H_8_N_2_)(H_2_O)]
*M* *_r_* = 793.13
Monoclinic, 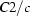

*a* = 28.3242 (5) Å
*b* = 6.2561 (2) Å
*c* = 23.6171 (4) Å
β = 119.539 (1)°
*V* = 3640.97 (15) Å^3^
*Z* = 4
Mo *K*α radiation
μ = 0.65 mm^−1^
*T* = 293 K
0.21 × 0.17 × 0.15 mm

#### Data collection

Rigaku R-AXIS RAPID diffractometer
Absorption correction: multi-scan (*ABSCOR*; Higashi, 1995 ▶) *T*_min_ = 0.875, *T*_max_ = 0.908
25489 measured reflections
4128 independent reflections
3904 reflections with *I* > 2σ(*I*)
*R*_int_ = 0.023

#### Refinement

*R*[*F* ^2^ > 2σ(*F* ^2^)] = 0.032
*wR*(*F* ^2^) = 0.083
*S* = 1.10
4128 reflections
246 parameters
1 restraint
H-atom parameters constrained
Δρ_max_ = 0.86 e Å^−3^
Δρ_min_ = −0.32 e Å^−3^

### 

Data collection: *RAPID-AUTO* (Rigaku, 1998 ▶); cell refinement: *RAPID-AUTO*; data reduction: *CrystalStructure* (Rigaku/MSC, 2002 ▶); program(s) used to solve structure: *SHELXS97* (Sheldrick, 2008 ▶); program(s) used to refine structure: *SHELXL97* (Sheldrick, 2008 ▶); molecular graphics: *X-SEED* (Barbour, 2001 ▶); software used to prepare material for publication: *publCIF* (Westrip, 2010 ▶).

## Supplementary Material

Crystal structure: contains datablocks global, I. DOI: 10.1107/S1600536811001280/hg2790sup1.cif

Structure factors: contains datablocks I. DOI: 10.1107/S1600536811001280/hg2790Isup2.hkl

Additional supplementary materials:  crystallographic information; 3D view; checkCIF report

## Figures and Tables

**Table 1:** Hydrogen-bond geometry (Å, °)

*D*—H⋯*A*	*D*—H	H⋯*A*	*D*⋯*A*	*D*—H⋯*A*
O1w—H1⋯O2^i^	0.84	2.09	2.741 (3)	135

Symmetry code: (i) 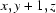.

**Fig. 1.**
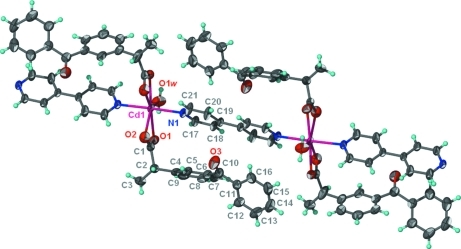
Thermal ellipsoid plot (Barbour, 2001) of fragment of the polymeric Cd(H2O)(C10H8N2)(C16H13O3)2 chain at the 50% probability level; hydrogen atoms are drawn as spheres of arbitrary radius. The disorder is not shown.

## supplementary crystallographic information

### Comment

The drug, 2-(3-benzoylphenyl)propanoic acid (Kétoprofène) (Briard & Rossi, 1990), forms a small number of metal derivatives; in the cobalt(II) and nickel(II) derivatives, the carboxyl unit binds in a unidentate manner to the water-coordinated metal atoms (Zhang *et al.*, 2007*a*, 2007*b*). In the title cadmium–4,4'-bipyridine adduct (Scheme I), the *N*-heterocycle links adjacent formula units into a chain running along the *c*-axis of the monoclinic unit cell. The cadmium atom, which lies on a twofold rotation axis, is chelated by the carboxyl unit and it exists in seven-coordinate pentagonal bipyramidal geometry. The apical sites are occupied by N atoms. The water molecule also lies on the twofold rotation axis (Fig. 1). The methyl subsituent of the carboxylate is disordered over two positions in a 1:1 ratio.

### Experimental

Cadmium nitrate (1 mmol) and 4,4'-bipyridine (1 mmol) were dissolved in ethanol (50 ml); the solution was added to an aqueous solution (50 ml) of 2-(3-benzoylphenyl)propanoic acid (2 mmol). The pH was adjusted to 7 by the addition of aqueous sodium hydroxide. The solution was filtered and then set aside for the growth of crystal.

### Refinement

The methyl substitutent in the carboxylate is disordered over two positions; as the occupancy refined to nearly 50%, the occupancy was then fixed as 50:50. The pair of C_methine_–C_methyl_ distances were restained to within 0.01 Å of each other.

All hydrogen atoms were generated geometrically (C–H 0.93 to 0.98, O–H 0.84; *U* 1.2 to 1.5 *U*_eq_ of the arrier atom).

### Crystal data

**Table:**

[Cd(C_16_H_13_O_3_)_2_(C_10_H_8_N_2_)(H_2_O)]	*F*(000) = 1624
*M**_r_* = 793.13	*D*_x_ = 1.447 Mg m^−^^3^
Monoclinic, *C*2/*c*	Mo *K*α radiation, λ = 0.71073 Å
Hall symbol: -C 2yc	Cell parameters from 24003 reflections
*a* = 28.3242 (5) Å	θ = 3.2–27.4°
*b* = 6.2561 (2) Å	µ = 0.65 mm^−^^1^
*c* = 23.6171 (4) Å	*T* = 293 K
β = 119.539 (1)°	Prism, colorless
*V* = 3640.97 (15) Å^3^	0.21 × 0.17 × 0.15 mm
*Z* = 4	

### Data collection

**Table:**

Rigaku R-AXIS RAPID diffractometer	4128 independent reflections
Radiation source: fine-focus sealed tube	3904 reflections with *I* > 2σ(*I*)
graphite	*R*_int_ = 0.023
Detector resolution: 10.000 pixels mm^-1^	θ_max_ = 27.4°, θ_min_ = 3.2°
ω scans	*h* = −36→33
Absorption correction: multi-scan (*ABSCOR*; Higashi, 1995)	*k* = −8→8
*T*_min_ = 0.875, *T*_max_ = 0.908	*l* = −29→30
25489 measured reflections	

### Refinement

**Table:**

Refinement on *F*^2^	Primary atom site location: structure-invariant direct methods
Least-squares matrix: full	Secondary atom site location: difference Fourier map
*R*[*F*^2^ > 2σ(*F*^2^)] = 0.032	Hydrogen site location: inferred from neighbouring sites
*wR*(*F*^2^) = 0.083	H-atom parameters constrained
*S* = 1.10	*w* = 1/[σ^2^(*F*_o_^2^) + (0.0418*P*)^2^ + 4.0713*P*] where *P* = (*F*_o_^2^ + 2*F*_c_^2^)/3
4128 reflections	(Δ/σ)_max_ = 0.001
246 parameters	Δρ_max_ = 0.86 e Å^−^^3^
1 restraint	Δρ_min_ = −0.32 e Å^−^^3^

### Fractional atomic coordinates and isotropic or equivalent isotropic displacement parameters (Å^2^)

**Table:**

	*x*	*y*	*z*	*U*_iso_*/*U*_eq_	Occ. (<1)
Cd1	0.5000	0.57510 (3)	0.7500	0.03727 (8)	
O1	0.40639 (7)	0.6179 (3)	0.67563 (9)	0.0607 (5)	
O2	0.43201 (9)	0.2876 (3)	0.70014 (11)	0.0704 (5)	
O3	0.40988 (11)	0.0777 (4)	0.45591 (12)	0.0850 (7)	
O1W	0.5000	0.9428 (4)	0.7500	0.0785 (10)	
H1	0.4679	0.9876	0.7303	0.118*	
N1	0.50515 (8)	0.5506 (3)	0.65299 (9)	0.0442 (4)	
C1	0.39770 (10)	0.4249 (5)	0.66638 (11)	0.0532 (6)	
C2	0.34256 (14)	0.3558 (9)	0.60990 (15)	0.1154 (18)	
H2	0.3500	0.2034	0.6086	0.138*	0.50
H2'	0.3186	0.4612	0.6136	0.138*	0.50
C3	0.3003 (2)	0.3399 (14)	0.6217 (3)	0.0763 (17)	0.50
H3A	0.3135	0.2957	0.6659	0.114*	0.50
H3B	0.2747	0.2366	0.5927	0.114*	0.50
H3C	0.2828	0.4764	0.6147	0.114*	0.50
C3'	0.3174 (3)	0.1758 (11)	0.6087 (3)	0.0765 (18)	0.50
H3'1	0.3217	0.1494	0.6511	0.115*	0.50
H3'2	0.3328	0.0595	0.5967	0.115*	0.50
H3'3	0.2795	0.1883	0.5775	0.115*	0.50
C4	0.33928 (11)	0.4258 (6)	0.54630 (13)	0.0717 (10)	
C5	0.35646 (10)	0.2915 (5)	0.51355 (11)	0.0600 (7)	
H5	0.3699	0.1568	0.5304	0.072*	
C6	0.35392 (10)	0.3555 (4)	0.45536 (11)	0.0512 (5)	
C7	0.33546 (11)	0.5580 (4)	0.43111 (13)	0.0561 (6)	
H7	0.3340	0.6025	0.3927	0.067*	
C8	0.31913 (12)	0.6943 (6)	0.46458 (15)	0.0712 (8)	
H8	0.3070	0.8312	0.4488	0.085*	
C9	0.32088 (12)	0.6274 (7)	0.52109 (16)	0.0799 (10)	
H9	0.3094	0.7198	0.5428	0.096*	
C10	0.37381 (11)	0.2037 (4)	0.42323 (12)	0.0561 (6)	
C11	0.34975 (11)	0.2039 (4)	0.35106 (12)	0.0523 (5)	
C12	0.29677 (12)	0.2666 (5)	0.30938 (14)	0.0625 (7)	
H12	0.2751	0.3145	0.3262	0.075*	
C13	0.27593 (15)	0.2578 (6)	0.24244 (15)	0.0805 (9)	
H13	0.2404	0.3000	0.2144	0.097*	
C14	0.30791 (18)	0.1868 (6)	0.21779 (16)	0.0863 (11)	
H14	0.2938	0.1803	0.1730	0.104*	
C15	0.36051 (17)	0.1253 (6)	0.25842 (18)	0.0837 (10)	
H15	0.3821	0.0796	0.2412	0.100*	
C16	0.38120 (13)	0.1315 (5)	0.32456 (15)	0.0671 (7)	
H16	0.4166	0.0868	0.3520	0.080*	
C17	0.47859 (10)	0.6901 (4)	0.60522 (10)	0.0496 (5)	
H17	0.4608	0.8029	0.6125	0.060*	
C18	0.47596 (10)	0.6764 (4)	0.54526 (10)	0.0492 (5)	
H18	0.4571	0.7794	0.5136	0.059*	
C19	0.50120 (9)	0.5111 (4)	0.53218 (9)	0.0419 (4)	
C20	0.52919 (13)	0.3651 (5)	0.58225 (12)	0.0653 (8)	
H20	0.5471	0.2503	0.5762	0.078*	
C21	0.53023 (13)	0.3917 (5)	0.64114 (12)	0.0634 (8)	
H21	0.5495	0.2932	0.6741	0.076*	

### Atomic displacement parameters (Å^2^)

**Table:**

	*U*^11^	*U*^22^	*U*^33^	*U*^12^	*U*^13^	*U*^23^
Cd1	0.04538 (13)	0.04118 (13)	0.02536 (11)	0.000	0.01752 (9)	0.000
O1	0.0547 (10)	0.0751 (14)	0.0467 (10)	0.0085 (9)	0.0207 (8)	−0.0110 (9)
O2	0.0836 (14)	0.0608 (13)	0.0684 (12)	−0.0055 (11)	0.0386 (11)	−0.0031 (10)
O3	0.1018 (17)	0.0817 (16)	0.0668 (14)	0.0390 (13)	0.0380 (13)	0.0248 (11)
O1W	0.0694 (18)	0.0446 (16)	0.093 (2)	0.000	0.0182 (18)	0.000
N1	0.0528 (10)	0.0534 (12)	0.0303 (8)	0.0050 (8)	0.0236 (8)	0.0042 (7)
C1	0.0472 (12)	0.082 (2)	0.0352 (11)	−0.0121 (12)	0.0238 (10)	−0.0136 (11)
C2	0.077 (2)	0.227 (5)	0.0424 (15)	−0.081 (3)	0.0299 (15)	−0.035 (2)
C3	0.046 (3)	0.108 (5)	0.070 (4)	−0.010 (3)	0.025 (3)	−0.004 (4)
C3'	0.087 (4)	0.084 (5)	0.058 (3)	−0.036 (4)	0.035 (3)	−0.007 (3)
C4	0.0467 (13)	0.121 (3)	0.0383 (13)	−0.0263 (16)	0.0142 (11)	−0.0198 (15)
C5	0.0533 (13)	0.0784 (19)	0.0374 (11)	−0.0092 (12)	0.0139 (10)	0.0032 (12)
C6	0.0487 (12)	0.0573 (14)	0.0383 (11)	−0.0031 (11)	0.0144 (9)	0.0004 (10)
C7	0.0565 (14)	0.0568 (16)	0.0402 (12)	−0.0005 (11)	0.0124 (10)	−0.0020 (10)
C8	0.0612 (15)	0.069 (2)	0.0603 (17)	0.0041 (14)	0.0122 (13)	−0.0176 (14)
C9	0.0555 (16)	0.114 (3)	0.0595 (18)	−0.0091 (17)	0.0202 (13)	−0.0391 (19)
C10	0.0635 (14)	0.0530 (15)	0.0495 (13)	0.0027 (12)	0.0260 (11)	0.0097 (11)
C11	0.0624 (14)	0.0463 (14)	0.0490 (13)	−0.0069 (11)	0.0282 (11)	0.0004 (10)
C12	0.0649 (15)	0.0590 (17)	0.0550 (14)	−0.0027 (12)	0.0228 (12)	−0.0084 (12)
C13	0.089 (2)	0.069 (2)	0.0538 (16)	−0.0030 (17)	0.0120 (15)	−0.0080 (14)
C14	0.124 (3)	0.082 (2)	0.0515 (16)	−0.014 (2)	0.0419 (19)	−0.0025 (16)
C15	0.107 (3)	0.093 (3)	0.076 (2)	−0.019 (2)	0.064 (2)	−0.0076 (19)
C16	0.0711 (17)	0.0711 (19)	0.0688 (18)	−0.0083 (14)	0.0420 (15)	−0.0003 (14)
C17	0.0615 (13)	0.0566 (15)	0.0361 (11)	0.0146 (11)	0.0282 (10)	0.0063 (10)
C18	0.0603 (13)	0.0560 (14)	0.0334 (10)	0.0153 (11)	0.0247 (10)	0.0113 (9)
C19	0.0490 (11)	0.0509 (12)	0.0291 (10)	0.0035 (9)	0.0217 (8)	0.0042 (9)
C20	0.101 (2)	0.0636 (17)	0.0439 (13)	0.0355 (16)	0.0457 (14)	0.0172 (12)
C21	0.096 (2)	0.0645 (17)	0.0384 (12)	0.0317 (15)	0.0397 (13)	0.0189 (11)

### Geometric parameters (Å, °)

**Table:**

Cd1—O1W	2.301 (3)	C6—C7	1.382 (4)
Cd1—O1^i^	2.3653 (18)	C6—C10	1.489 (4)
Cd1—O1	2.3653 (18)	C7—C8	1.388 (4)
Cd1—N1	2.3713 (17)	C7—H7	0.9300
Cd1—N1^i^	2.3713 (17)	C8—C9	1.376 (5)
Cd1—O2	2.469 (2)	C8—H8	0.9300
Cd1—O2^i^	2.469 (2)	C9—H9	0.9300
O1—C1	1.230 (3)	C10—C11	1.491 (3)
O2—C1	1.247 (4)	C11—C12	1.385 (4)
O3—C10	1.218 (3)	C11—C16	1.393 (4)
O1W—H1	0.8400	C12—C13	1.390 (4)
N1—C17	1.328 (3)	C12—H12	0.9300
N1—C21	1.329 (3)	C13—C14	1.370 (5)
C1—C2	1.534 (4)	C13—H13	0.9300
C2—C3'	1.325 (6)	C14—C15	1.371 (6)
C2—C3	1.360 (6)	C14—H14	0.9300
C2—C4	1.523 (4)	C15—C16	1.372 (4)
C2—H2	0.9800	C15—H15	0.9300
C2—H2'	0.9800	C16—H16	0.9300
C3—H3A	0.9600	C17—C18	1.384 (3)
C3—H3B	0.9600	C17—H17	0.9300
C3—H3C	0.9600	C18—C19	1.376 (3)
C3'—H3'1	0.9600	C18—H18	0.9300
C3'—H3'2	0.9600	C19—C20	1.392 (3)
C3'—H3'3	0.9600	C19—C19^ii^	1.494 (4)
C4—C9	1.382 (5)	C20—C21	1.387 (3)
C4—C5	1.383 (4)	C20—H20	0.9300
C5—C6	1.399 (3)	C21—H21	0.9300
C5—H5	0.9300		
			
O1W—Cd1—O1^i^	83.50 (5)	C2—C3'—H3'3	109.5
O1W—Cd1—O1	83.50 (5)	H3'1—C3'—H3'3	109.5
O1^i^—Cd1—O1	167.01 (11)	H3'2—C3'—H3'3	109.5
O1W—Cd1—N1	93.71 (5)	C9—C4—C5	118.4 (3)
O1^i^—Cd1—N1	98.30 (7)	C9—C4—C2	120.9 (4)
O1—Cd1—N1	82.55 (7)	C5—C4—C2	120.7 (4)
O1W—Cd1—N1^i^	93.71 (5)	C4—C5—C6	120.9 (3)
O1^i^—Cd1—N1^i^	82.55 (7)	C4—C5—H5	119.5
O1—Cd1—N1^i^	98.30 (7)	C6—C5—H5	119.5
N1—Cd1—N1^i^	172.58 (10)	C7—C6—C5	119.7 (3)
O1W—Cd1—O2	136.77 (6)	C7—C6—C10	122.3 (2)
O1^i^—Cd1—O2	139.63 (8)	C5—C6—C10	118.0 (3)
O1—Cd1—O2	53.34 (8)	C6—C7—C8	119.4 (3)
N1—Cd1—O2	84.10 (7)	C6—C7—H7	120.3
N1^i^—Cd1—O2	90.49 (7)	C8—C7—H7	120.3
O1W—Cd1—O2^i^	136.77 (6)	C9—C8—C7	120.2 (3)
O1^i^—Cd1—O2^i^	53.34 (8)	C9—C8—H8	119.9
O1—Cd1—O2^i^	139.63 (8)	C7—C8—H8	119.9
N1—Cd1—O2^i^	90.49 (7)	C8—C9—C4	121.4 (3)
N1^i^—Cd1—O2^i^	84.10 (7)	C8—C9—H9	119.3
O2—Cd1—O2^i^	86.47 (11)	C4—C9—H9	119.3
C1—O1—Cd1	94.23 (16)	O3—C10—C6	120.0 (2)
C1—O2—Cd1	88.96 (17)	O3—C10—C11	119.5 (3)
Cd1—O1W—H1	109.5	C6—C10—C11	120.5 (2)
C17—N1—C21	116.76 (19)	C12—C11—C16	118.8 (3)
C17—N1—Cd1	119.96 (15)	C12—C11—C10	122.5 (2)
C21—N1—Cd1	123.07 (15)	C16—C11—C10	118.7 (3)
O1—C1—O2	122.6 (2)	C11—C12—C13	120.1 (3)
O1—C1—C2	117.4 (3)	C11—C12—H12	120.0
O2—C1—C2	120.0 (3)	C13—C12—H12	120.0
O1—C1—Cd1	59.23 (13)	C14—C13—C12	119.8 (3)
O2—C1—Cd1	64.04 (14)	C14—C13—H13	120.1
C2—C1—Cd1	169.56 (19)	C12—C13—H13	120.1
C3'—C2—C3	54.9 (5)	C13—C14—C15	120.8 (3)
C3'—C2—C4	116.9 (4)	C13—C14—H14	119.6
C3—C2—C4	126.2 (4)	C15—C14—H14	119.6
C3'—C2—C1	124.3 (5)	C14—C15—C16	119.7 (3)
C3—C2—C1	117.7 (4)	C14—C15—H15	120.1
C4—C2—C1	108.5 (2)	C16—C15—H15	120.1
C3'—C2—H2	45.1	C15—C16—C11	120.8 (3)
C3—C2—H2	99.2	C15—C16—H16	119.6
C4—C2—H2	99.2	C11—C16—H16	119.6
C1—C2—H2	99.2	N1—C17—C18	123.4 (2)
C3'—C2—H2'	100.7	N1—C17—H17	118.3
C3—C2—H2'	46.6	C18—C17—H17	118.3
C4—C2—H2'	100.7	C19—C18—C17	120.3 (2)
C1—C2—H2'	100.7	C19—C18—H18	119.9
H2—C2—H2'	145.7	C17—C18—H18	119.9
C2—C3—H3A	109.5	C18—C19—C20	116.36 (19)
C2—C3—H3B	109.5	C18—C19—C19^ii^	122.0 (3)
H3A—C3—H3B	109.5	C20—C19—C19^ii^	121.6 (3)
C2—C3—H3C	109.5	C21—C20—C19	119.7 (2)
H3A—C3—H3C	109.5	C21—C20—H20	120.2
H3B—C3—H3C	109.5	C19—C20—H20	120.2
C2—C3'—H3'1	109.5	N1—C21—C20	123.5 (2)
C2—C3'—H3'2	109.5	N1—C21—H21	118.3
H3'1—C3'—H3'2	109.5	C20—C21—H21	118.3
			
O1W—Cd1—O1—C1	177.46 (15)	Cd1—C1—C2—C4	−2.5 (18)
O1^i^—Cd1—O1—C1	177.46 (15)	C3'—C2—C4—C9	126.0 (6)
N1—Cd1—O1—C1	82.84 (15)	C3—C2—C4—C9	61.3 (7)
N1^i^—Cd1—O1—C1	−89.72 (15)	C1—C2—C4—C9	−87.2 (4)
O2—Cd1—O1—C1	−5.33 (14)	C3'—C2—C4—C5	−56.3 (6)
O2^i^—Cd1—O1—C1	0.9 (2)	C3—C2—C4—C5	−120.9 (7)
C1^i^—Cd1—O1—C1	−7.4 (4)	C1—C2—C4—C5	90.5 (4)
O1W—Cd1—O2—C1	9.29 (18)	C9—C4—C5—C6	−1.7 (4)
O1^i^—Cd1—O2—C1	−175.73 (14)	C2—C4—C5—C6	−179.5 (2)
O1—Cd1—O2—C1	5.24 (14)	C4—C5—C6—C7	1.9 (4)
N1—Cd1—O2—C1	−79.84 (15)	C4—C5—C6—C10	179.2 (2)
N1^i^—Cd1—O2—C1	105.24 (15)	C5—C6—C7—C8	−0.7 (4)
O2^i^—Cd1—O2—C1	−170.71 (18)	C10—C6—C7—C8	−177.9 (3)
C1^i^—Cd1—O2—C1	−175.28 (10)	C6—C7—C8—C9	−0.6 (4)
O1W—Cd1—N1—C17	−37.10 (19)	C7—C8—C9—C4	0.7 (5)
O1^i^—Cd1—N1—C17	−121.1 (2)	C5—C4—C9—C8	0.4 (4)
O1—Cd1—N1—C17	45.85 (19)	C2—C4—C9—C8	178.2 (3)
O2—Cd1—N1—C17	99.6 (2)	C7—C6—C10—O3	147.3 (3)
O2^i^—Cd1—N1—C17	−174.0 (2)	C5—C6—C10—O3	−29.9 (4)
O1W—Cd1—N1—C21	148.4 (2)	C7—C6—C10—C11	−32.9 (4)
O1^i^—Cd1—N1—C21	64.5 (2)	C5—C6—C10—C11	149.9 (2)
O1—Cd1—N1—C21	−128.6 (2)	O3—C10—C11—C12	151.6 (3)
O2—Cd1—N1—C21	−74.9 (2)	C6—C10—C11—C12	−28.2 (4)
O2^i^—Cd1—N1—C21	11.5 (2)	O3—C10—C11—C16	−26.2 (4)
C1—Cd1—N1—C21	−101.9 (2)	C6—C10—C11—C16	154.0 (3)
C1^i^—Cd1—N1—C21	37.7 (2)	C16—C11—C12—C13	−0.4 (4)
Cd1—O1—C1—O2	10.1 (3)	C10—C11—C12—C13	−178.1 (3)
Cd1—O1—C1—C2	−168.7 (2)	C11—C12—C13—C14	0.1 (5)
Cd1—O2—C1—O1	−9.6 (3)	C12—C13—C14—C15	−0.4 (6)
Cd1—O2—C1—C2	169.1 (2)	C13—C14—C15—C16	1.1 (6)
O1W—Cd1—C1—O1	−2.68 (16)	C14—C15—C16—C11	−1.4 (5)
O1^i^—Cd1—C1—O1	−177.57 (14)	C12—C11—C16—C15	1.0 (5)
N1—Cd1—C1—O1	−93.06 (15)	C10—C11—C16—C15	178.9 (3)
N1^i^—Cd1—C1—O1	93.98 (15)	C21—N1—C17—C18	0.2 (4)
O2—Cd1—C1—O1	170.6 (2)	Cd1—N1—C17—C18	−174.6 (2)
O2^i^—Cd1—C1—O1	−179.35 (14)	N1—C17—C18—C19	0.6 (4)
O1—C1—C2—C3'	−150.3 (5)	C17—C18—C19—C20	−0.8 (4)
O2—C1—C2—C3'	30.9 (6)	C17—C18—C19—C19^ii^	179.3 (3)
Cd1—C1—C2—C3'	141.2 (14)	C18—C19—C20—C21	0.2 (5)
O1—C1—C2—C3	−85.7 (6)	C19^ii^—C19—C20—C21	−179.9 (3)
O2—C1—C2—C3	95.5 (6)	C17—N1—C21—C20	−0.9 (5)
Cd1—C1—C2—C3	−154.1 (13)	Cd1—N1—C21—C20	173.8 (3)
O1—C1—C2—C4	65.9 (4)	C19—C20—C21—N1	0.7 (5)
O2—C1—C2—C4	−112.9 (4)		

Symmetry codes: (i) −*x*+1, *y*, −*z*+3/2; (ii) −*x*+1, −*y*+1, −*z*+1.

### Hydrogen-bond geometry (Å, °)

**Table:**

*D*—H···*A*	*D*—H	H···*A*	*D*···*A*	*D*—H···*A*
O1w—H1···O2^iii^	0.84	2.09	2.741 (3)	135

Symmetry codes: (iii) *x*, *y*+1, *z*.
